# Cardiovascular magnetic resonance of total and atrial pericardial adipose tissue: a validation study and development of a 3 dimensional pericardial adipose tissue model

**DOI:** 10.1186/1532-429X-15-73

**Published:** 2013-08-29

**Authors:** Rajiv Mahajan, Pawel Kuklik, Suchi Grover, Anthony G Brooks, Christopher X Wong, Prashanthan Sanders, Joseph B Selvanayagam

**Affiliations:** 1 Centre for Heart Rhythm Disorders (CHRD), University of Adelaide and Royal Adelaide Hospital, Adelaide, Australia; 2 Discipline of Medicine, Flinders University and Flinders Medical Centre, Bedford Park, Australia

**Keywords:** Atrial, Pericardial adipose tissue, Validation, CMR

## Abstract

**Background:**

Recently pericardial adipose tissue (PAT) has been shown to be an independent predictor of atrial fibrillation (AF). Atrial PAT may influence underlying atrial musculature creating a substrate for AF. This study sought to validate the assessment of total and atrial PAT by standard cardiovascular magnetic resonance (CMR) measures and describe and validate a three dimensional atrial PAT model.

**Methods:**

10 merino cross sheep underwent CMR using a 1.5 Tesla system (Siemens, Sonata, Erlangen, Germany). Atrial and ventricular short axis (SA) images were acquired, using ECG -gated steady state free precession sequences. In order to quantify total volume of adipose tissue, a three dimensional model was constructed from consecutive end-diastolic images using semi-automated software. Regions of adipose tissue were marked in each slice followed by linear interpolation of pixel intensities in spaces between consecutive image slices. Total volume of adipose tissue was calculated as a total volume of the three dimensional model and the mass estimated from volume measurements. The sheep were euthanized and pericardial adipose tissue was removed and weighed for comparison to the corresponding CMR measurements.

**Results:**

All CMR adipose tissue estimates significantly correlated with autopsy measurements (ICC > 0.80; p < 0.03). Intra- observer reliability in CMR measures was high, with 95% levels of agreement within 5.5% (ICC = 0.995) for total fat mass and its individual atrial (95% CI ± 8.3%, ICC = 0.993) and ventricular components (95% CI ± 6.6%, ICC = 0.989). Inter- observer 95% limits of agreement were within ± 10.7% (ICC = 0.979), 7.4% (ICC = 0.991) and 7.2% (ICC = 0.991) for atrial, ventricular and total pericardial adipose tissue, respectively.

**Conclusion:**

This study validates the use of a semi-automated three dimensional atrial PAT model utilizing standard (clinical) CMR sequences for accurate and reproducible assessment of atrial PAT. The measurement of local cardiac fat stores via this methodology could provide a sensitive tool to examine the regional effect of fat deposition on atrial substrate which potentially may influence AF ablation strategies in obese patients.

## Background

The increasing prevalence of atrial fibrillation (AF) has been in-part attributed to the aging population and its associated co-morbidities such as hypertension, coronary artery disease and heart failure. However, there is increasing evidence that obesity contributes to the burden of atrial fibrillation in our population. Analyses from the Framingham and Women Health Studies (WHS) suggest that obesity is associated with an increased risk of AF independent of other traditional risk factors [1,2]. These population-based studies have utilized body mass index as a measure of obesity. Recently, pericardial adipose tissue (PAT), both total and atrial, has been demonstrated to predict AF risk independently of weight and other measures of obesity [3-5]. This has two important implications. Firstly, PAT could have a causal relationship with the risk of AF and it has been hypothesized that this effect could be mediated locally [6-8]. Secondly, a change in atrial PAT mass with weight change could be a better predictor of the risk of AF as compared to other measures of weight change. Although, ventricular paracardiac adipose tissue CMR measurement has been validated before, the validation of the patchy and smaller volume of atrial PAT with the gold standard of mass at autopsy has not been previously performed [9]. In this study we sought to validate the quantification of atrial and total PAT utilizing a three dimensional atrial PAT model.

## Methods

Ten merino cross sheep with weight ranging from 67 to 103 kilograms were included in the study. All procedures were conducted in accordance with the guidelines outlined in the “Position of the American Heart Association on Research Animal Use” adopted on November 11, 1984 by the American Heart Association. The study protocol was reviewed and approved by the Animal Research Ethics Committees of University of Adelaide and Institute of Medical and Veterinary Services, Adelaide, Australia.

### Definitions

The fat deposits around the heart have been defined in this study as:

○ *Epicardial adipose tissue* was defined as the adipose tissue between the myocardium and visceral pericardium [10,11].

○ *Paracardiac adipose tissue* was defined as the adipose tissue adherent and external to the parietal pericardium [12].

○ *Pericardial adipose* tissue was defined the sum of the epicardial and paracardiac adipose tissue [13].

### Cardiovascular magnetic resonance protocol and analysis

All animals underwent cardiovascular magnetic resonance (CMR) immediately prior to euthanasia for the evaluation of pericardial adipose tissue. CMR was performed using a 1.5 Tesla system (Siemens, Sonata, Erlangen, Germany). The animals were anaesthetized and placed supine within the CMR bore. The animals were mechanically ventilated to allow breath holding sequences to be performed. Sequential steady state free precession (SSFP) short-axis cine sequences were acquired with 6 mm slice-thickness and no interslice gaps through both the atrial and the ventricles. Slices were aligned to at 90 degrees to the long axis of the left ventricle, and planned from the most cranial aspect of the left atrium and proceeded caudally to the left ventricular apex. All images were acquired at end expiration with the following parameters (slice thickness 6 mm/0 mm, TR/TE 52.05 ms/1.74 ms, flip angle 70 degree, matrix 256 × 150, FOV 380 mm). In order to replicate standard clinical protocols, this study intentionally did not incorporate adipose-specific sequences.

**Figure 1:**
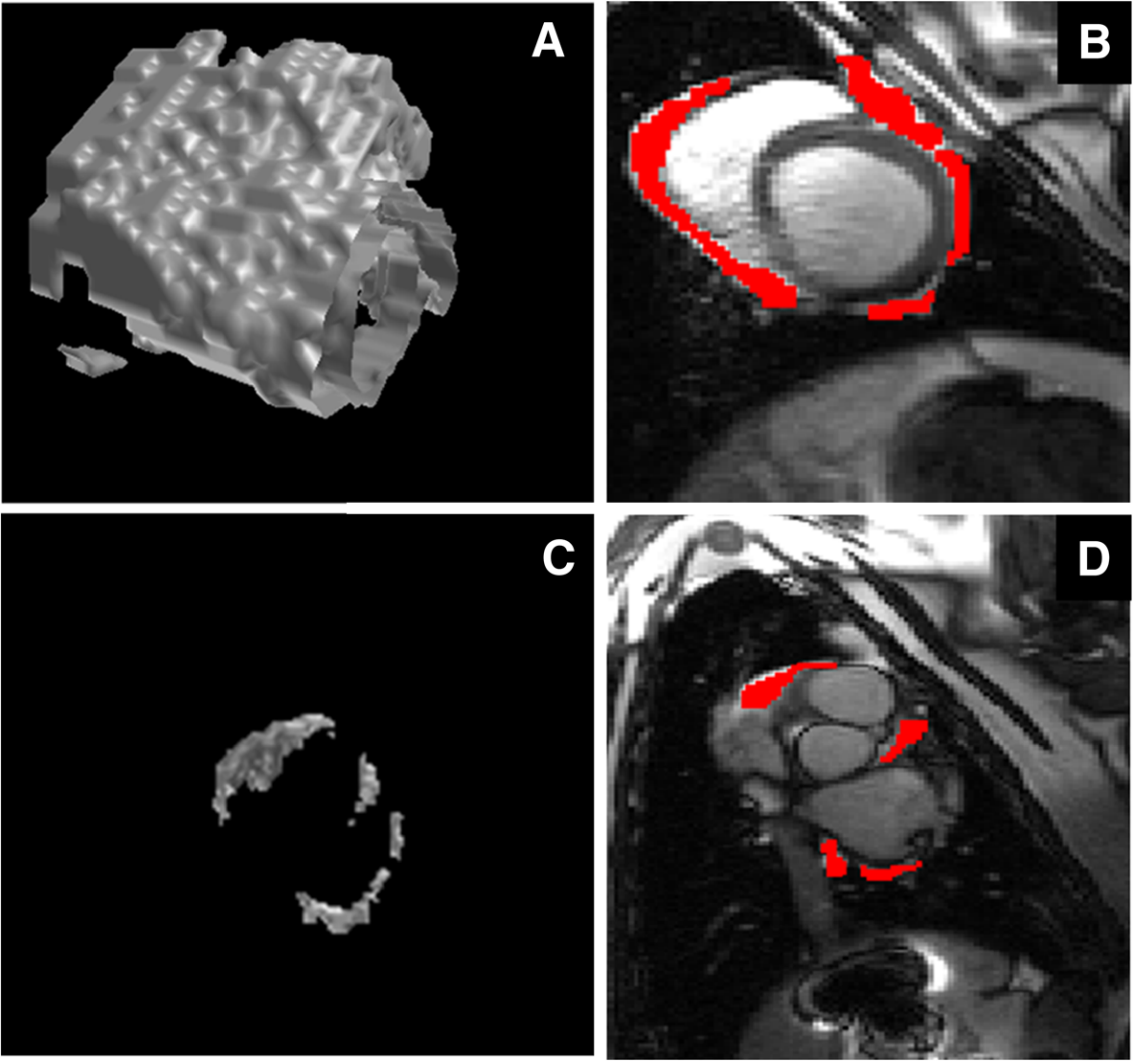
**Three dimensional model of pericardial adipose tissue using semi-automated in-house software.** Adipose tissue was marked on each slice followed by interpolation of pixel intensities between consecutive slices. **A)** A three dimensional rendered model of the ventricular pericardial adipose tissue. **B)** Short axis view of the left and right ventricles with the pericardial adipose tissue marked in red. **C)** A three dimensional model of the atrial pericardial adipose tissue. **D)** A short axis view of the left and right atrium with the pericardial adipose tissue marked in red.

The image analysis was conducted offline using custom made software. The areas of pericardial adipose tissue were traced on consecutive end diastolic images (Figure 1). The ventricular pericardial adipose tissue was defined as adipose tissue from the mitral valve hinge (point of insertion at the annulus) down to the ventricular apex, inclusive of the most inferior margin of the adipose tissue. The atrial pericardial adipose tissue was defined as the pericardial adipose tissue lying above the mitral valve hinge and below the right pulmonary artery. Not all CMR images were of sufficient quality to adequately define the visceral pericardium, particularly in atrial slices, and as a result no attempt was made to differentiate epicardial and paracardiac components of adipose tissue.

In order to quantify total volume of adipose tissue, a three dimensional (3D) model was constructed using semi-automated software developed by the authors. Regions of PAT were marked in each slice followed by linear interpolation of pixel intensities in spaces between consecutive image slices. The total fat volume was quantified on the three dimensional model was converted to mass by multiplying it with a density constant of 0.9.

Assessment of atria, ventricular and total cardiac fat volume measured by CMR scans was performed twice by the primary investigator to determine intra-observer measurement error. An additional independent and experienced investigator also measured the fat volume components to establish an inter-observer agreement. All measures of CMR fat were performed blinded to the autopsy values.

### Pericardial fat quantification at autopsy

**Figure 2:**
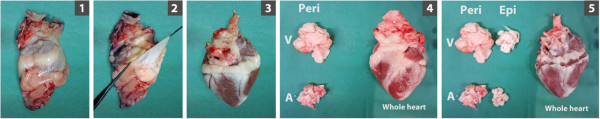
**The process of atrial and ventricular pericardial adipose tissue measurement via autopsy.** Steps **1)** Removal of heart with pulmonary veins, pulmonary artery and part of trachea, **2)** Pericardium incised along the atrio-ventricular (AV) groove and was peeled off inferiorly to the ventricular apex to harvest paracardiac ventricular adipose tissue and superiorly to harvest atrial paracardiac adipose tissue, **3)** Posterior view of the heart after removal of paracardiac adipose tissue, with epicardial atrial adipose tissue visible posterior (adjacent to posterior LA wall) and laterally along the AV groove, **4)** Anterior view of the heart with atrial and ventricular paracardiac adipose tissue to the left. Atrial paracardiac adipose tissue was located between the appendages and the great vessels, with little fat deposition on the appendages, **5)** Posterior view of the heart after dissecting the majority of epicardial adipose tissue.

The sheep were euthanized following acquisition of CMR images. While the sheep were under general anesthesia, the heart was surgically removed from the sheep thorax, with particular attention paid to retrieving all the pericardial adipose tissue. To ensure that all pericardial fat was collected, the heart was excised with the pulmonary veins, right pulmonary artery and the trachea intact. An incision was made circumferentially along the atrio-ventricular groove and the pericardium peeled back towards the apex (Figure 2; Step 2) and collected as the paracardiac ventricular fat. The remaining pericardium was peeled upwards toward the pulmonary artery with all fat up to the pulmonary artery branches being assigned to paracardiac atrial fat. After the trachea was removed from the heart, the fat was then meticulously dissected from the ventricle and atria to collect the epicardial fat (Figure 2; Step 4). The total pericardial adipose tissue for the atria and ventricles was calculated as the sum of paracardiac and epicardial components (Figure 2; Step 5).

### Statistical analysis

Normally distributed continuous data are reported as mean ± standard deviation. The agreement between autopsy total, atrial and ventricular pericardial adipose tissue masses and those derived from CMR were assessed via intra-class correlation coefficients (ICC) and Bland and Altman plots with 95% limits of agreement. Consistency, rather than absolute ICCs were calculated via a two way random effects model because of the expected systematic bias toward lower masses on autopsy due to technical limitations in removing all fat from the ex-vivo heart. The inter- and intra-observer reliability of CMR fat assessment was assessed using the same methodology, except that consistency ICCs were calculated. All tests were performed using PASW (Version 18 IBM, Armonk, NY) with p < 0.05 deemed significant.

## Results

Ten sheep with a mean weight of 80 ± 8 kg were assessed for both CMR and autopsy pericardial adipose tissue assessment, however one sheep was excluded from further analyses due to CMR artifacts precluding accurate assessment. Mean CMR imaging time was 30 minutes per animal. The additional mean time duration to assess pericardial adipose tissue with the semi-automated software was 5 ± 1.4 minutes per animal. On CMR assessment atrial, ventricular and total adipose tissue were assessed as 37 ± 10, 250 ± 71, 287 ± 77 g, respectively. On autopsy, the corresponding values were 29 ± 8, 231 ± 70, 260 ± 74 g. Table 1 shows the pericardial adipose tissue CMR measures and the corresponding autopsy values.

**Table 1:** Pericardial adipose tissue (PAT) mass as assessed on Autopsy and CMR

**Sheep**	**Total PAT-autopsy (gms)**	**Ventricular PAT- autopsy (gms)**	**Atrial PAT-autopsy (gms)**	**Total PAT-CMR (gms)**	**Ventricular PAT- CMR (gms)**	**Atrial PAT- CMR (gms)**
1	235	195	40	279	232	47
2	285	265	20	302	273	29
3	414	372	42	446	401	45
4	298	268	30	320	277	42
5	194	172	22	210	184	26
6	253	225	28	267	239	28
7	216	182	34	257	207	50
8	286	260	26	320	279	41
9	160	140	20	177	155	23

### Autopsy pericardial adipose tissue regional distribution

The atrial epicardial adipose tissue was distributed predominately adjacent to posterior wall of left atrium and adjacent to the atrio-ventricular groove. The atrial paracardiac adipose tissue deposits were mainly between the appendages and the great arteries (Figure 2). The ventricular epicardial adipose tissue was distributed predominately along the atrio-ventricular groove and the coronary arteries. In contrast, the paracardiac ventricular fat was evenly distributed over the surface of the pericardium.

#### Agreement of CMR assessment with Autopsy measures of pericardial adipose tissue

**Figure 3:**
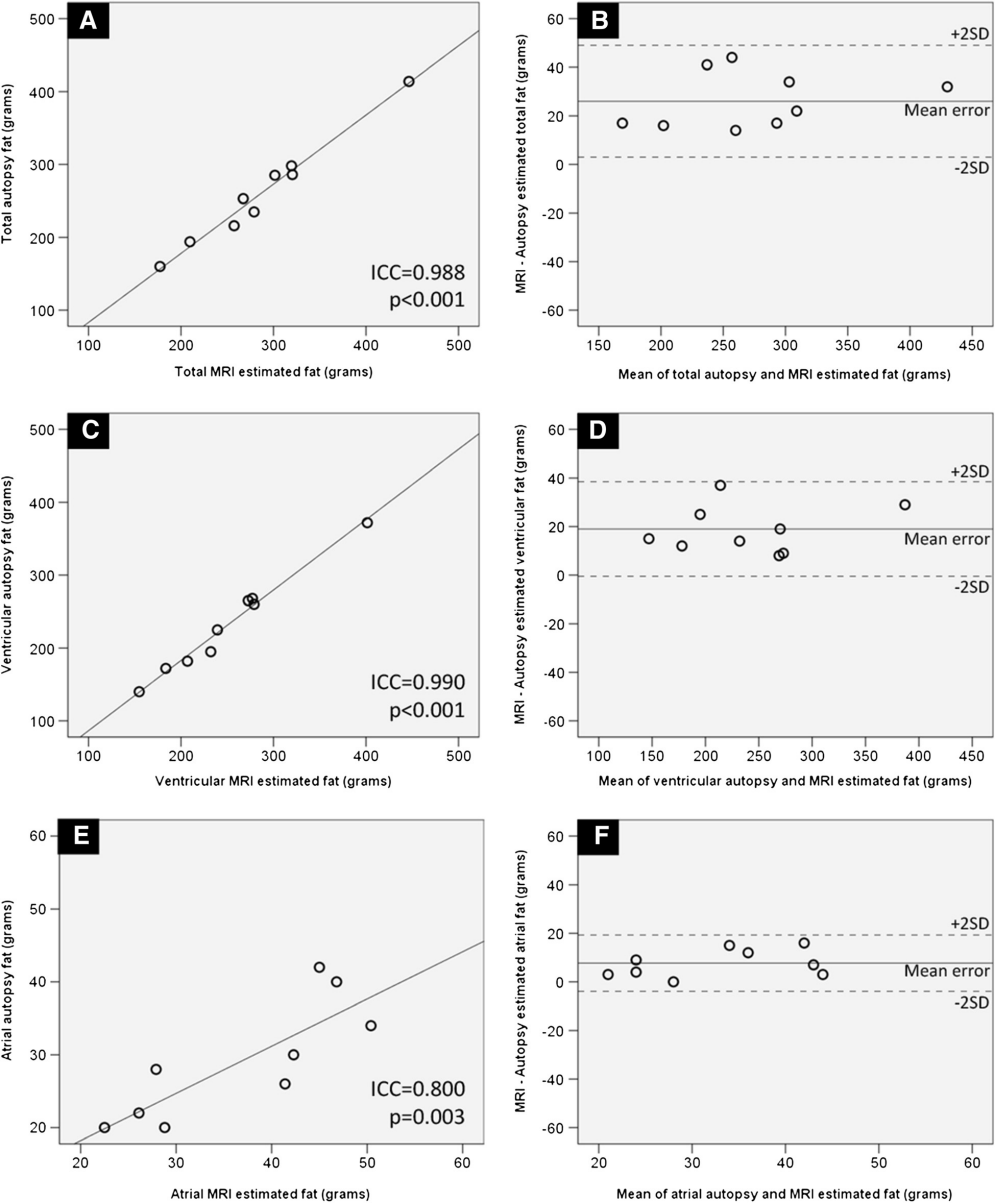
**Agreement of CMR assessment with autopsy measures of pericardial adipose tissue.** Panel **A** shows the limits of agreement (ICC) between total pericardial adipose tissue measures on Autopsy and CMR. Panel **B** represents the degree of agreement, via Bland and Altman plots. Similarly, the panels **C**, **D** and **E**, **F** represent limits of agreement and Bland and Altman plots for ventricular and atrial pericardial adipose tissue, respectively.

Atrial, ventricular and total CMR pericardial adipose tissue correlated strongly with autopsy measurements (ICC > 0.80; p < 0.003) (Figure 3). CMR systematically over-estimated total autopsy fat by a mean of 26 g, within 95% confidence limits of ± 23.0 g. The corresponding ventricular and atrial components were overestimated by a mean of 19.0 g (95% CI ± 19.5 gms) and 7.7 g (95% CI ± 11.6 gms), respectively.

#### Intra- and inter-observer reliability of CMR assessment of pericardial adipose tissue

Intra- observer reliability for CMR measured atrial, ventricular and total fat mass assessment measures was high (ICC > 0.993), with 95% levels of agreement ± 5.5% for total fat mass, ± 8.3% for atrial fat mass and ± 6.6% for ventricular mass (Table 2). Similarly, all components of fat measurement (total, ventricular and atrial) ICCs exceeded 0.991 for inter- observer reliability. The 95% limits of agreement were within ± 10.7%, ± 7.4% and ± 7.2% for atrial, ventricular and total pericardial adipose tissue, respectively (Table 3).

**Table 2:** Intra-observer reproducibility of CMR measures of pericardial adipose tissue (PAT)

**PAT**	**Intra-observer 95% CI ( gms)**	**Intra-observer 95% CI (%)**	**p value**
**Total**	15.9	5.5	0.001
**Ventricular**	16.4	6.6	0.001
**Atrial**	3.1	8.3	0.003

**Table 3:** Inter-observer reproducibility of CMR measures of pericardial adipose tissue (PAT)

**PAT**	**Inter- observer 95% CI (gms)**	**Inter- observer 95% CI (%)**	**p value**
**Total**	20.9	7.2	0.001
**Ventricular**	18.6	7.4	0.001
**Atrial**	3.9	10.7	0.001

## Discussion

To the best of our knowledge, this is the first study to validate a CMR technique to specifically assess atrial pericardial adipose tissue. Our findings demonstrate that: [1] CMR assessment of atrial, ventricular and total fat has high intra- and inter-observer reproducibility, and [2] CMR quantified atrial and ventricular adipose tissue strongly agrees with ex vivo adipose mass, validating its use as a robust measure of pericardial adiposity. Moreover, the findings were reproducible and the image sequences utilized were those which would normally be acquired during a standard clinical CMR scan. Importantly, this study validates a three dimensional model of atrial pericardial adipose tissue which can be utilized to study regional fat deposits with respect to underlying atrial electrophysiological properties.

Echocardiography and computed tomography have previously been utilized for pericardial fat assessment. However, echocardiography is limited by its inability to obtain pericardial volumes and often sub-optimal imaging windows [14]. Moreover, echocardiography has been limited to assessment of ventricular pericardial adipose tissue. Although, volumetric assessment of pericardial adipose tissue can be performed by CT, the exposure to ionizing radiation limits its use, especially if serial measurements are planned [15].

Obesity has been demonstrated, in several population based studies, to be a novel risk factor for atrial fibrillation [16-19]. Its association with hypertension, obstructive sleep apnea, diabetes mellitus, coronary artery disease and congestive heart failure, clouds interpretation of the potential mechanisms by which obesity is linked to atrial fibrillation. Population level studies have suggested that the effect may be mediated through left atrial enlargement [16,17], albeit evidence is mounting that pericardial adipose tissue, surrounding the heart, may also play a significant role in the development of atrial fibrillation. Batal et al suggested that computed-tomography (CT) measured pericardial fat thickness is associated with prevalent AF [5]. In participants of the Framingham Heart Study, a community-based cohort, *Thanassoulis et al* observed that higher pericardial fat volumes were associated with a nearly 40% higher odds of prevalent AF [3]. Wong et al have recently demonstrated that pericardial fat volume is a better predictor than body mass index in predicting severity of atrial fibrillation and even outcomes after ablation [4]. As adipose tissue is highly vascular, it is hypothesized that mediators of lipid metabolism and inflammation produced in the pericardial adipose tissue may affect the underlying atrial myocardium in a paracrine manner [8]. In addition, epicardial fat has been demonstrated to infiltrate underlying atrial musculature in an obese ovine model [20]. This fatty infiltration can mechanically separate muscle fibers and create an area of electrical silence promoting reentry and atrial fibrillation. A recent study has reported the association of elevated dominant frequency with epicardial fat location [21].

This study demonstrates that CMR assessment of pericardial fat is an accurate and reproducible measure that could be utilized in future larger clinical cohorts. Furthermore, three dimensional PAT model constructed from CMR slices, allows regional interpretation of local cardiac fat stores, that in future could be utilized in conjunction with electro-anatomic maps to investigate the atrial substrate for atrial fibrillation. This could potentially have implications for planning ablation strategies for atrial fibrillation ablation in obese individuals.

Ventricular paracardiac adipose tissue has previously been validated using CMR [9]. This current study, to the best of our knowledge, is the first to validate CMR measure of atrial pericardial fat. Furthermore, in the study by Nelson et al [9], the paracardiac adipose tissue was validated against the histological gold standard. In contrast, in the current study, the total pericardial fat volume was validated, as the epicardial adipose tissue was meticulously removed along with the paracardiac adipose tissue. This provided a more accurate comparison between autopsy and CMR pericardial adipose tissue measures. However, complete removal of epicardial adipose tissue was not possible due to its adherence to underlying myocardium in some cases, and this could be a reason for a small systematic over-estimation by CMR (Figure 3).

### Limitations

Fat suppression sequences were intentionally not performed in the study as our intention was to validate measurement of atrial pericardial adipose tissue using a standard clinical CMR protocol. However, fat suppression sequences could improve adjudication of adipose tissue and further improve inter- and intraobserver variability. Furthermore, although stringent attempts were made to completely remove all epicardial adipose tissue at autopsy, this was not always possible.

### Clinical implications

With the increasing incidence and burden of atrial fibrillation, the identification and characterization of new risk factors is of public health importance [22]. Atrial PAT has been demonstrated to predict AF risk independent of other measures of obesity. Given the dual epidemics of obesity and atrial fibrillation, non-invasive CMR measurement of atrial PAT could provide incremental information on the risk of developing AF. Moreover, atrial pericardial adipose tissue could provide insights in evaluating the variability of AF risk in obese individuals with change in weight [2]. The three dimensional model of pericardial fat validated in this study could be utilized to investigate the regional effect of pericardial fat on underlying atrial myocardium. In addition, the information on regional interaction of pericardial fat with adjacent atrial tissue may provide new insights for planning ablation strategies for atrial fibrillation in obese individuals.

## Conclusion

CMR measurement of atrial, ventricular and total pericardial adipose tissue via 3D modeling of fat stores is a reproducible and accurate measure of pericardial fat on autopsy. The measurement of local cardiac fat stores via this methodology could provide a more sensitive tool to examine the potential causal relationship between obesity and atrial fibrillation and examine the regional effect of fat deposition on atrial substrate.

## Competing interests

Dr. Mahajan is supported by the Australian Postgraduate Award and Leo J. Mahar Electrophysiology Scholarships from the University of Adelaide. Dr Grover is supported by the MF and MH Joyner Scholarship in Medicine from the Flinders University. Dr. Wong is supported by a Rhodes Scholarship and a Postgraduate Scholarship from the National Health and Medical Research Council of Australia. Drs. Brooks, Kuklik, Sanders are funded by the National Heart Foundation of Australia. Dr Sanders is also supported by the Practitioner Fellowship from the National Health and Medical Research Council of Australia.

Dr. Sanders reports having served on the advisory board of St. Jude Medical, Bard Electrophysiology, Biosense-Webster, Medtronic, Sanofi-Aventis, and Merck. Dr. Sanders reports having received lecture fees from St. Jude Medical, Bard Electrophysiology, Biosense-Webster, Medtronic and Merck. Dr. Sanders reports having received research funding from St. Jude Medical, Bard Electrophysiology, Biosense-Webster and Medtronic. Dr Selvanayagam reports having received consulting fees from Kai Pharmaceuticals, Phillips, Siemens and Bristol Myers Sqiubb. Dr. Selvanayagam reports having received lecture fees from St. Jude Medical, Medtronic, Siemens, Pfizer, Bayer and AstraZenecca. He reports having received research funding from Siemens.

## Authors’ contributions

RM and PS originated the idea for the study. PK designed the three dimensional pericardial fat software. RM, PS and JBS were responsible for the design and execution of the analysis. RM, PK and SG were responsible for acquisition of data. RM, PK and JBS contributed to analysis of data. RM, PK, SG, AGB, CXW, PS and JBS were responsible for the clinical interpretation of the data. RM, PS and JBS were responsible for writing the manuscript. All authors had full access to and take full responsibility for the integrity of the data. All authors have read and approved to the manuscript as written.

## Authors’ information

Joseph B. Selvanayagam and Prashanthan Sanders are senior authors.
